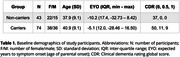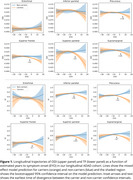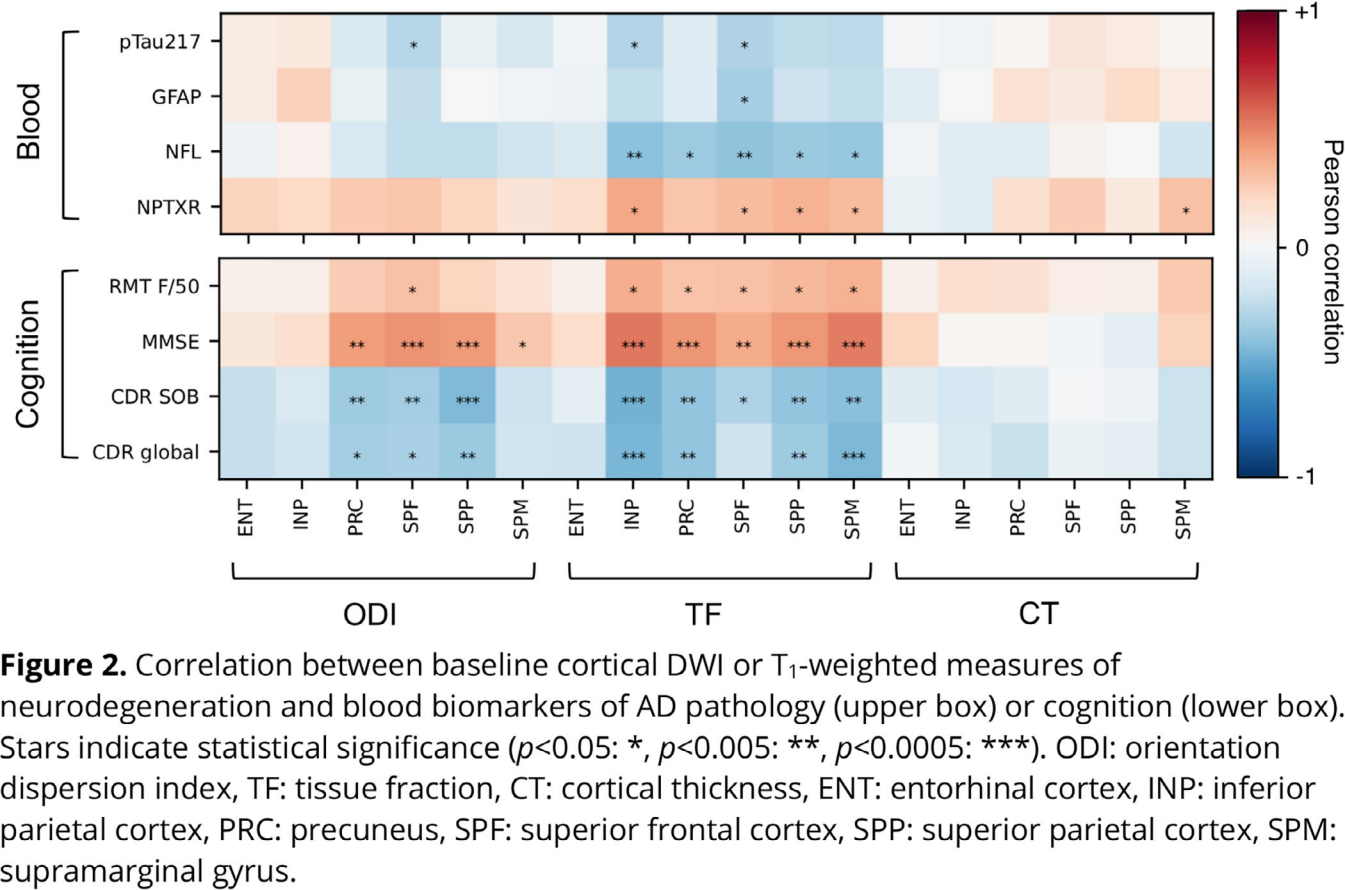# Longitudinal trajectories of advanced cortical diffusion‐weighted imaging measures of tissue microstructure in pre‐symptomatic autosomal dominant Alzheimer’s disease

**DOI:** 10.1002/alz70861_108939

**Published:** 2025-12-23

**Authors:** Christopher S Parker, Tom Veale, Ian B. Malone, Christopher Belder, Antoinette O'Connor, Emily Abel, Damien Ferguson, Amanda J Heslegrave, Hui Zhang, Nick C Fox, David M Cash, Philip SJ Weston

**Affiliations:** ^1^ UCL Hawkes Institute and Department of Computer Science, University College London, London UK; ^2^ Dementia Research Centre, Queen Square Institute of Neurology, London, Greater London UK; ^3^ UCL Dementia Research Centre, London UK; ^4^ Dementia Research Centre, UCL Queen Square Institute of Neurology, University College London, London UK; ^5^ UCL Dementia Research Centre, London, Greater London UK; ^6^ Dementia Research Centre, UCL Queen Square Institute of Neurology, London UK; ^7^ Dementia Research Centre, UCL Queen Square Institute of Neurology, London, London UK; ^8^ UK Dementia Research Institute at UCL, London UK; ^9^ UCL Hawkes Institute, London UK

## Abstract

**Background:**

In Alzheimer’s disease, quantitative biomarkers of early subtle neurodegeneration are needed to identify individuals at greatest risk of imminent cognitive decline. Advanced cortical diffusion‐weighted imaging (DWI) probes biologically specific tissue microstructural features, which may enable earlier detection of neurodegeneration than macrostructural measures (e.g., cortical thickness). Previous studies have examined neurodegeneration in symptomatic AD cross‐sectionally. Here, we analyse a longitudinal cohort of individuals with autosomal dominant AD (ADAD), spanning the full disease spectrum, to explore changes in advanced cortical DWI metrics throughout the pre‐symptomatic period.

**Method:**

74 autosomal ADAD mutation carriers and 49 non‐carrier siblings underwent annual (mean visits per participant = 2.85, Table 1) multi‐shell DWI, T_1_‐weighted imaging, plasma collection and neuropsychological testing. Region‐averaged advanced cortical DWI measures of orientation dispersion index (ODI), a proxy measure of dendritic complexity, and the intra‐voxel neuronal tissue fraction (TF); and cortical thickness, were extracted for 6 cortical regions previously reported to undergo pre‐symptomatic cortical thinning in ADAD. Longitudinal trajectories were characterised with mixed effects models. Associations with plasma biomarkers of pathology and cognitive measures were assessed with Pearson correlations adjusted for estimated years to symptom onset (EYO).

**Result:**

Longitudinal models show widespread significant associations between EYO and advanced cortical DWI microstructure metrics of ODI and TF in all regions in mutation carriers (*p* <0.05). Carrier temporal trajectories diverged from non‐carriers up to 3.7 years before symptom onset (Figure 1). No associations were found in non‐carriers. Significant associations were observed between TF and plasma markers of neuronal damage (NFL) and synaptic integrity (NPTXR); and with tests of memory and cognition (Figure 2). Cortical thickness (CT) associations were weaker and less consistent.

**Conclusion:**

Longitudinal analysis shows pre‐symptomatic changes in advanced cortical DWI metrics, suggesting sensitivity to early neurodegeneration. Regional differences in TF and ODI trajectories highlight potential spatiotemporal variation in microstructural breakdown. Associations between TF and plasma markers suggest close correspondence between this measure and early neuronal and synaptic loss. Stronger associations between cortical DWI measures and EYO, plasma and neuropsychological test scores, compared to cortical thickness measures, demonstrate the potential added value of advanced quantitative microstructure imaging for the early detection and characterisation of AD neurodegeneration.